# Differential expression and clinical significance of three inflammation-related microRNAs in gangliogliomas

**DOI:** 10.1186/s12974-015-0315-7

**Published:** 2015-05-20

**Authors:** A. S. Prabowo, J. van Scheppingen, A. M. Iyer, J. J. Anink, W. G. M. Spliet, P. C. van Rijen, A. Y. N. Schouten-van Meeteren, E. Aronica

**Affiliations:** Department of (Neuro)Pathology, Academic Medical Center, University of Amsterdam, Meibergdreef 9, 1105 AZ Amsterdam, The Netherlands; Department of Pediatric Oncology, Emma Children’s Hospital, Academic Medical Center, University of Amsterdam, Meibergdreef 9, 1105 AZ Amsterdam, The Netherlands; Department of Pathology, Rudolf Magnus Institute for Neuroscience, University Medical Center Utrecht, Utrecht, The Netherlands; Department of Neurosurgery, Rudolf Magnus Institute for Neuroscience, University Medical Center Utrecht, Utrecht, The Netherlands; SEIN - Stichting Epilepsie Instellingen Nederland, Heemstede, The Netherlands; Swammerdam Institute for Life Sciences, Center for Neuroscience, University of Amsterdam, Amsterdam, The Netherlands

**Keywords:** Gangliogliomas, miRNA, Real-time polymerase chain reactions, In situ hybridization, Immunohistochemistry, Inflammation, Epilepsy

## Abstract

**Purpose:**

miR21, miR146, and miR155 represent a trio of microRNAs which has been shown to play a key role in the regulation of immune and inflammatory responses. In the present study, we investigated the differential expression and clinical significance of these three miRNAs in glioneuronal tumors (gangliogliomas, GGs) which are characterized by prominent activation of the innate immune response.

**Methods:**

The expression levels of miR21, miR146, and miR155 were evaluated using Taqman PCR in 34 GGs, including 15 cases with sufficient amount of perilesional cortex. Their expression was correlated with the tumor features and the clinical history of epilepsy. In addition, in situ hybridization was used to evaluate their cellular distribution in both tumor and peritumoral cortex.

**Results:**

Increased expression of miR146a was observed in both tumor and peritumoral cortex compared to control samples. miR146a was detected in both neuronal and astroglial cells. Tumor and peritumoral miR146a expression was negatively correlated with frequency of seizures and the density of activated microglial cells. Neuronal and astroglial expression was observed for both miR21 and miR155 with increased expression of miR21 within the tumor and miR155 in the peritumoral region. Negative correlations were observed between the miRNA levels and the expression of putative targets within the astroglial component of the tumor.

**Conclusion:**

We report a differential regulation of three miRNAs, known to be related to inflammation, in both tumor and peritumoral cortex of patients with GG. Moreover, our findings suggest a functional relationship between miR146a expression and epilepsy, either directly in epileptogenesis or as modulation of seizure activity.

**Electronic supplementary material:**

The online version of this article (doi:10.1186/s12974-015-0315-7) contains supplementary material, which is available to authorized users.

## Introduction

Gangliogliomas (GGs) represent the most frequent tumor entity in young patients who undergo surgery for chronic intractable focal epilepsy [1, 2]. They are low-grade, slowly growing, cortically based tumors included within the group of long-term epilepsy associated tumor (LEAT; [2]). GGs display a very low risk for tumor recurrence and malignant progression. Although surgical intervention shows favorable prognosis, both in terms of tumor management and improving seizures, in a proportion of cases, seizures may persist despite surgery [1–4]. Resection of tumor alone has been associated with a less satisfactory outcome supporting the epileptogenic contribution of the peritumoral zone [5, 6]. Histologically, GGs are characterized by a mixture of dysmorphic neurons and glial tumor cells. Recent studies have provided evidence of a sustained inflammatory reaction in glioneuronal lesions with activation of both the innate and adaptive immune response and involvement of different inflammatory pathways, including the interleukin-1 receptor/Toll-like receptor pathway (IL-1R/TLR) [7–10]. Interestingly, experimental evidence indicates proconvulsant and ictogenic properties of these pathways, supporting the role of inflammation in the pathophysiology of human epilepsy (for review see [11–14]). Accordingly, it has also been shown that the density of activated microglia, as well as the number of IL-1β-positive neuronal cells in focal cortical dysplasia (FCD) and in GG was positively correlated with the frequency of seizures prior to surgical resection [7, 15, 16].

In recent years, microRNAs (miRNAs) have been reported as key post-transcriptional regulators of gene expression in several biological processes of the central nervous system, as well as in the pathogenesis of different neurological diseases and in oncogenesis [17–20]. Recently, both clinical and experimental studies have shown the potential contribution of miRNAs to epilepsy pathophysiology [21, 22]. miRNA array profiling studies point to the role of microRNAs involved in inflammatory processes [23–26]. During epileptogenesis in experimental temporal lobe epilepsy [26], a significant upregulation of miRNAs involved in the regulation of the IL-1R/TLR proinflammatory pathway, including miR146a, miR21, and miR155 [27–32], has been observed. Interestingly, miR146a has been shown to be induced in response to inflammatory cues as a negative-feedback regulator of the human astrocyte-mediated inflammatory response [25].

In the present study, we investigated the expression and cellular distribution of miR21, miR146, and miR155, three miRNAs involved in the regulation of inflammatory pathways with proictogenic properties [30], in a large cohort of gangliogliomas with well-characterized intractable epilepsy. In addition, we analyzed the expression of these miRNAs in the perilesional tissue, which is of particular interest for its possible contribution to generation/propagation of seizures. To provide better insights into the mechanisms underlying the intrinsic and high epileptogenicity of these glioneuronal lesions, we evaluated a possible relationship between changes in expression of these miRNAs, microglial activation, and the clinical course of epilepsy.

## Material and methods

### Subjects

The cases included in this study were obtained from the archives of the Department of Neuropathology of the Academic Medical Center (AMC, University of Amsterdam) and the University Medical Center in Utrecht (UMCU). A total of 34 brain tissue specimens, removed from patients undergoing surgery for intractable epilepsy, were examined. Tissue was obtained and used in accordance with the Declaration of Helsinki and the AMC Research Code provided by the Medical Ethics Committee and approved by the science committee of the UMC Utrecht Biobank. All cases were reviewed independently by two neuropathologists, and the diagnosis was confirmed according to the revised WHO classification of tumors of the central nervous system [33]. Thirty-two patients underwent resection of the tumor for medically intractable epilepsy. The predominant type of seizure pattern was that of complex partial seizures, which were resistant to maximal doses of anti-epileptic drugs (AEDs). The patients with epilepsy underwent presurgical evaluation [34], and the post-operative seizure outcome was classified according to Engel [35]. Twenty patients underwent tailored temporal lobe epilepsy surgery. The clinical features of the included GG patients are summarized in Table 1. We included 15 GGs cases that contained sufficient amount of peritumoral tissue (normal-appearing cortex/white matter adjacent to the tumor), for comparison with the autopsy specimens. Control cortex/white matter from the temporal region was obtained at autopsy from eight adult control patients without history of neurological diseases (years/range, 25–52; female/male (F/M), 4/4). Autopsy brain tissues from patients with neuro-inflammatory pathologies (viral encephalitis; herpes simplex encephalitis and rabies encephalitis) were also examined as positive controls. All autopsies were performed within 12 h after death. Furthermore, we also used histologically normal temporal neocortex from three male adult patients (F/M, 2/1; years/range, 20–32) undergoing extensive surgical resection of the mesial structures for the treatment of medically intractable complex partial epilepsy.

**Table 1:** Summary of clinical findings of GGs

Patient	Gender	Localization	Age of surgery (year)	Age of seizure onset (year)	Duration of epilepsy (year)	Pre-operative seizure frequency last month^d^	Post-operative outcome (Engel’s score)
1^a,c^	F	T	29	13	16	200	1a
2^a,c^	M	T	19	10	9	20	1a
3^a,c^	M	T	17	14	3	25	1a
4^a,c^	F	T	11	8	3	56	1a
5^a,c^	M	T	16	14	2	30	1a
6^a,c^	M	T	24	1	23	100	1a
7^a,c^	F	T	42	11	31	60	1a
8^a,c^	M	T	31	12	19	150	1a
9^a,b,c^	M	T	21	13	8	70	2a
10^a,c^	F	T	6	4	2	30	1a
11^a,c^	F	T	14	11	3	55	1a
12^a,c^	M	T	17	15	2	25	1a
13^a,b,c^	F	P	24	23	1	3	1b
14^a,c^	F	T	23	14	9	60	1a
15^a,c^	F	T	19	15	4	70	1a
16^a^	M	T	18	13	5	20	1a
17^a,b^	M	P	28	27	1	80	1b
18^a^	M	T	3	1	2	70	1a
19^a^	F	T	34	11	23	90	1a
20^a^	F	T	0.5	1	0.5	60	3a
21^a^	M	T	17	1	16	100	1a
22^a^	M	T	20	11	9	160	1a
23^a^	F	Fr	2	0. 25	2	70	1b
24^a^	F	T	31	22	9	40	1a
25^a^	F	T	33	8	25	90	1a
26^a^	F	T	49	19	30	150	NA
27^b^	F	T	22	10	12	20	1a
28^b^	M	T	34	16	18	10	1a
29^b^	M	T	33	18	15	50	1a
30^b^	F	T	56	52	4	60	2a
31^b^	F	T	28	12	16	80	2a
32^b^	F	Cer	48	No seizures	–	–	–
33^b^	M	P	16	11	5	60	1b
34^b^	F	BS	29	No seizures	–	–	–

*M* male, *F* female, *Fr* frontal, *T* temporal, *P* parietal, *Cer* cerebellum, *BS* brain stem

^a^FFPE (formalin-fixed, paraffin-embedded) tissue

^b^Frozen tissue

^c^Peritumoral cortex tissue

^d^Pre-operative seizure frequency

### Tissue preparation

Brain tissue from the control autopsy patients (*n* = 8) and surgical tissue block from patients with GG were snap frozen in liquid nitrogen and stored at −80 °C until further use (RNA isolation for RT-PCR). Additional tissue was fixed in 10 % buffered formalin and embedded in paraffin. Paraffin-embedded tissue was sectioned at 5 μm, mounted on pre-coated glass slides (Star Frost, Waldemar Knittel GmbH, Brunschweig, Germany), and used for in situ hybridizations and immunocytochemistry, as described below. One representative paraffin block per case were sectioned, stained, and assessed. Sections of all specimens were processed for hematoxylin and eosin (HE), as well as for immunocytochemical stainings for a number of neuronal and glial markers to confirm the diagnosis of ganglioglioma.

### In situ hybridization

In situ hybridization (ISH) for miR21, miR146a, and miR155 were performed using a 5′ - 3′ fluorescein (FAM) and double digoxygenin (DIG)-labeled Superior probes (miR21; DIG-TcaAcaTcaGucTgaTaaGcuA-DIG; miR146a: FAM-AacCcaTggAauTcaGuuCucA; miR155: DIG-AccCcuAucAcgAuuAgcAuuAa-DIG; Ribotask ApS, Odense, Denmark). The hybridizations were done on 5 μm sections of paraffin-embedded materials as described previously [26]. The probes were hybridized at 53 °C (miR21) and 56 °C (miR146a and miR155) for 1 h, and the hybridization was detected with alkaline phosphatase (AP)-labeled anti-DIG (Roche Applied Science, Basel, Switzerland) and AP-labeled anti-fluorescein (Roche Applied Science, Basel, Switzerland). NBT (nitro-blue tetrazolium chloride)/BCIP (5-bromo-4-chloro-3′-indolyphosphate p-toluidine salt) was used as chromogenic substrate for AP. Negative control assays were performed without probes and without primary antibody (sections were blank). For the double-staining, combining immunocytochemistry with in situ hybridization, the sections were first processed for ISH and then processed for immunocytochemistry with glial fibrillary acidic protein (GFAP; monoclonal mouse, Sigma, St. Louis, Mo, USA; 1:4000), NeuN (neuronal nuclear protein; mouse clone MAB377; Chemicon, Temecula, CA, USA; 1:2000), (HLA)-DP, DQ, DR (mouse clone CR3/43; DAKO, Glostrup, Denmark; 1:400), or CD34 (mouse clone QBEnd10; Immunotech, Marseille, Cedex, France; 1:600). Signal was detected using the chromogen 3-amino-9-ethylcarbazole (Sigma-Aldrich, St. Louis, MO, USA).

### RNA isolation

For RNA isolation, frozen material or cell culture material was homogenized in Qiazol Lysis Reagent (Qiagen Benelux, Venlo, The Netherlands). The total RNA including the miRNA fraction was isolated using the miRNeasy Mini kit (Qiagen Benelux, Venlo, the Netherlands) according to manufacturer’s instructions. The concentration and purity of RNA were determined at 260/280 nm using a Nanodrop spectrophotometer (Ocean Optics, Dunedin, FL, USA). Formalin-fixed paraffin-embedded (FFPE) material was processed for RNA isolation using QuickExtract™ FFPE (RNA Extraction Kit (Epicentre, Madison, WI, USA) according to manufacturer’s instructions. The concentration of RNA was determined using Qubit® 2.0 Fluorometer (Life Technologies, Carlsbad, CA, USA).

### Real-time quantitative PCR analysis (qPCR)

miRNA (miR21, miR146a, miR155, and the U6B small nuclear RNA gene, Rnu6B; miR23a) expression was analyzed using Taqman microRNA assays (Applied Biosystems, Foster City, CA). cDNA was generated using Taqman MicroRNA reverse transcription kit (Applied Biosystems, Foster City, CA) according to the manufacturer’s instructions, and the PCRs were run on a Roche Lightcycler 480 thermo cycler (Roche Applied Science, Basel, Switzerland).

Quantification of data was performed using the computer program LinRegPCR in which linear regression on the Log (fluorescence) per cycle number data is applied to determine the amplification efficiency per sample [36, 37]. The starting concentration of each specific product was divided by the starting concentration of reference gene (Rnu6B or miR23a), and this ratio was compared between groups.

To evaluate the microRNA targets (IRAK1, IRAK2, TNF receptor associated factor 6 (TRAF6), programmed cell death 4 (PDCD4), SHIP1, ERBB4, MEF2C, NOTCH1, NUMB, and phosphatase and tensin homolog (PTEN)), five micrograms of total RNA (*n* = 10 GG; *n* = 8 controls) were reverse-transcribed into cDNA using oligo dT primers (see Additional file 1: Table S1).

### Immunohistochemistry

Single-label immunohistochemistry (see Table 2) was performed, as previously described [10, 38]. The sections were deparaffinated in xylene, rinsed in ethanol (100 %, 95 %, 70 %), and incubated for 20 min in 0.3 % hydrogen peroxide diluted in methanol. Antigen retrieval was performed using a pressure cooker in 0.1 M citrate buffer pH 6.0 at 120 °C for 10 min. Slides were washed with phosphate-buffered saline (PBS; 0.1 M, pH 7.4) and incubated overnight with the primary antibody in PBS at 4 °C. Hereafter, the sections were washed in PBS and stained with a polymer-based peroxidase immunocytochemistry detection kit (PowerVision Peroxidase system, ImmunoVision, Brisbane, CA, USA). After washing, the sections were stained with 3,3′-diaminobenzidine tetrahydrochloride (50 mg DAB, Sigma-Aldrich, Zwijndrecht, The Netherlands) and 5 μl 30 % hydrogen peroxide in a 10-ml solution of Tris–HCl. The sections were counterstained with hematoxylin, dehydrated in alcohol and xylene, and coverslipped. The sections incubated without primary antibodies or with pre-immune serum were essentially blank. The sections were counterstained with hematoxylin. Immunostaining for BRAF V600E (clone VE1, Spring Bioscience, Pleasanton, CA, USA; [39]) was performed on a Ventana BenchMark XT immunostainer (Ventana Medical Systems, Tucson, AZ, USA).

**Table 2:** Immunohistochemistry: primary antibodies

Antigen	Primary Antibody	Source	Dilution
Glial fibrillary acidic protein (GFAP)	Polyclonal rabbit	DAKO, Glostrup, Denmark	1:4,000
Neuronal nuclear protein (NeuN)	Mouse clone MAB377	Chemicon, Temecula, CA, USA	1:2,000
Synaptophysin	Mouse clone Sy38	DAKO Glostrup, Denmark	1:200
Ki67	Mouse clone MIB-1	DAKO, Glostrup, Denmark	1:200
BRAF V600E	VE1 clone	–^a^	1:5
CD34	Mouse clone QBEnd10	Immunotech, Marseille, Cedex, France	1:600
Phospho-S6 ribosomal protein (pS6)	Monoclonal rabbit (Ser235/236)	Cell Signaling Technology, Beverly, MA, USA	1:200
Human leukocyte antigen (HLA-DP, DQ, DR; MHC-II)	Mouse clone CR3/43	DAKO, Glostrup, Denmark	1:100
IL-1β	Polyclonal goat antibody	Santa Cruz Bio., CA, USA	1:70
IRAK1	Mouse clone 3 F7	Sigma, St. Louis, Mo, USA,	1:300
TRAF6	Monoclonal rabbit	Abcam, Cambridge, MA, USA	1:300
PDCD4	Polyclonal rabbit	Abcam, Cambridge, MA, USA	1:450
SHIP1	Polyclonal rabbit	Cell Signaling, Beverly, MA, USA	1:300

*MHC* major histocompatibility complex

^a^Kindly provided by D. Capper and A. von Deimling [39]

### Evaluation of histology and immunohistochemistry

All labeled tissue sections were evaluated by two independent observers blinded to clinical data for the presence or absence of various histopathological parameters and specific immunoreactivity (IR) for the different markers. Hematoxylin–eosin (HE) stained slides were used to evaluate the neuronal and glial components of the tumors, the presence of dysplastic neurons, calcifications, and perivascular cuffs of lymphocytes. We also semi-quantitatively evaluated the IR for the different markers, such as synaptophysin, GFAP, CD34, and HLA-DR (MHC-II). The intensity of IL-1β immunoreactive staining was evaluated using a scale of 0–3 (0: −, no; 1: +/−, weak; 2: +, moderate; 3: ++, strong staining). All areas of the tumors, as well the peritumoral cortex, were examined, and the score represents the predominant cell staining intensity found in each case. The frequency of IL-1β, TRAF6, IRAK1, PDCD4, and SHIP1-positive cells [(1) rare, (2) sparse, (3) high] was also evaluated to give information about the relative number of positive glial cells within and around the tumor. As proposed before [7, 40], the product of these two values (intensity and frequency scores) was taken to give the overall score (total score; total score; immunoreactivity score; IRS). The numbers of HLA-DR-positive microglia/macrophages were quantified as previously described [15, 41].

### Cell cultures

The astrocytoma cell line U373 was obtained from the American Type Culture Collection (Rockville, MD, USA); cells were cultured in Dulbecco’s Modified Eagle’s Medium (DMEM)/HAM F10 (1:1) supplemented with 50 units/ml penicillin, 50 μg/ml streptomycin, and 10 % FCS on poly-l-lysine-coated plates.

For astrocytes-enriched human cell cultures, fetal brain tissue (15–20 weeks of gestation) was obtained from spontaneous or medically induced abortions with appropriate maternal written consent for brain autopsy. Tissue was obtained in accordance with the Declaration of Helsinki and the AMC Research Code provided by the Medical Ethics Committee of the AMC. Resected tissue samples were collected in DMEM/HAM F10 (1:1) medium (Gibco, Life Technologies), supplemented with 50 units/ml penicillin and 50 μg/ml streptomycin and 10 % fetal calf serum (FCS). Cell isolation was performed as previously described [25].

### Treatment of cell cultures

Human recombinant (r)IL-1β (Peprotech, NJ, USA; 10 ng/ml) was applied and maintained for 24 h before harvesting the cells for RNA isolation, as previously shown [42], the viability of human astrocytes in culture was not influenced by the treatments.

### Statistical analysis

Statistical analyses were performed with SPSS for Windows (SPSS 20, SPSS Inc., Chicago, IL, USA). Continuous variables were described with mean and ranges; categorical variables with proportions and percentages. The two-tailed Student’s *t* test or the non-parametric Kruskal–Wallis test followed by the Dunn’s post hoc test was used to assess differences between the groups. Correlation between immunohistochemical and clinical features (duration of epilepsy, seizure frequency, age at surgery, age at seizure onset, epilepsy outcome, etc.) were assessed using the Spearman’s rank correlation test. A value of *p* < 0.05 was defined as statistically significant.

## Results

### Case material and histological features

The clinical features of the cases included in this study are summarized in Table 1. Among the 34 patients (15 males and 19 females) included in the study, 32 had a history of chronic pharmacoresistant epilepsy. Postoperatively, 30 out of 32 patients with epilepsy were completely seizure free (Engel’s class I). Thirty-two had supratentorial tumors (*n* = 28 in temporal lobe). The mean age at the time of surgery was 23.9 years (range 1–56; SD 12.9). The mean age at epilepsy onset was 13.2 years (range 0.25–52; SD 9.5); the mean pre-operative seizure frequency was 67.6 per month (range 3–200; SD 45.9); and the mean duration of epilepsy was 10.5 years (range 0.5–31, SD 9). BRAF V600E mutation was evaluated in 33 GGs and detected in 15 cases (45.4 %).

### miR21, miR146a, and miR155 expression by real-time qPCR in GG and peritumoral cortex

miR21, miR146a, and miR155 expression was studied using qPCR in control human cerebral cortex samples and in GG samples. As previously shown for miR146a [25], there were no significant differences in expression between autopsy and surgical control samples, and miRNA expression levels between paired frozen and FFPE control samples were similar (not shown).

**Fig. 1:**
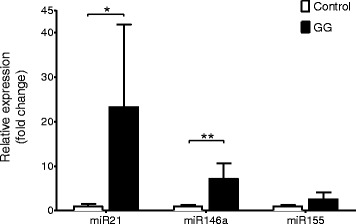
Quantitative real-time PCR of miR21, miR146a, and miR155 in GG. Expression levels of miR21, miR146a, and miR155 in GG (*n* = 9; frozen material). Data are expressed relative to the levels observed in control cortex (*n* = 8, frozen material); miRNA expression was normalized to that of the U6B small nuclear RNA gene (Rnu6B). The error bars represent SEM; statistical significance: *p < 0.05; **p < 0.01

**Fig. 2:**
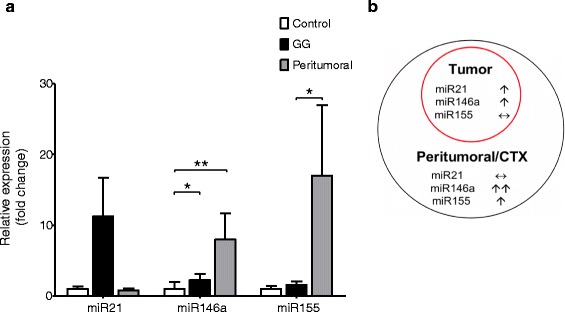
miR21, miR146a, and miR155 expression levels in GG and peritumoral cortex. **a** Quantitative real-time PCR of miR21, miR146a, and miR155 in GG (*n* = 26; formalin-fixed, paraffin-embedded, FFPE material) and peritumoral cortex (ctx; *n* = 15; FFPE material). Data are expressed relative to the levels observed in control ctx (*n* = 5, FFPE); miRNAs expression was normalized to that of the U6B small nuclear RNA gene (Rnu6B). The error bars represent SEM; statistical significance: *p < 0.05; **p < 0.01. **b** Schematic representation of miR21, miR146a, and miR155 changes in GG and peritumoral ctx

The expression of miR21, miR146a, and miR155 was evaluated in both the frozen (Fig. 1; *n* = 9) and FFPE (Fig. 2; *n* = 26) GG samples. Recent studies indicate a robust stability of miRNAs, supporting the accuracy of miRNA measurements with RT-qPCR also in FFPE tissues [43, 44].

Expression of miR21 in frozen tissues was significantly increased in GG compared to control cortex (*p* = 0.02; Fig. 1). Evaluation of miR21 in a larger cohort (FFPE tissue; including 15 cases with perilesional cortex) showed a tendency towards increased expression compared to both control and peritumoral cortex (*p* = 0.5; Fig. 2).

Expression of miR146a in frozen tissue was significantly higher in GG compared to control cortex (*p* = 0.002; Fig. 1). Increased expression of miR146a was observed in both the tumor and peritumoral cortex compared to the control samples (FFPE material; *p* = 0.01; Fig. 2). There was no significant difference in the expression of miR146a between GG and peritumoral cortex.

Expression of miR155 in both frozen and FFPE tissue showed no difference between GG and control cortex (*p* = 0.1; Figs. 1 and 2). However, miR155 was highly expressed in peritumoral cortex, showing a significant difference compared to GG (*p* = 0.01; Fig. 2).

### miR21, miR146a, and miR155 cellular distribution by in situ hybridization in GG and peritumoral cortex

**Fig. 3:**
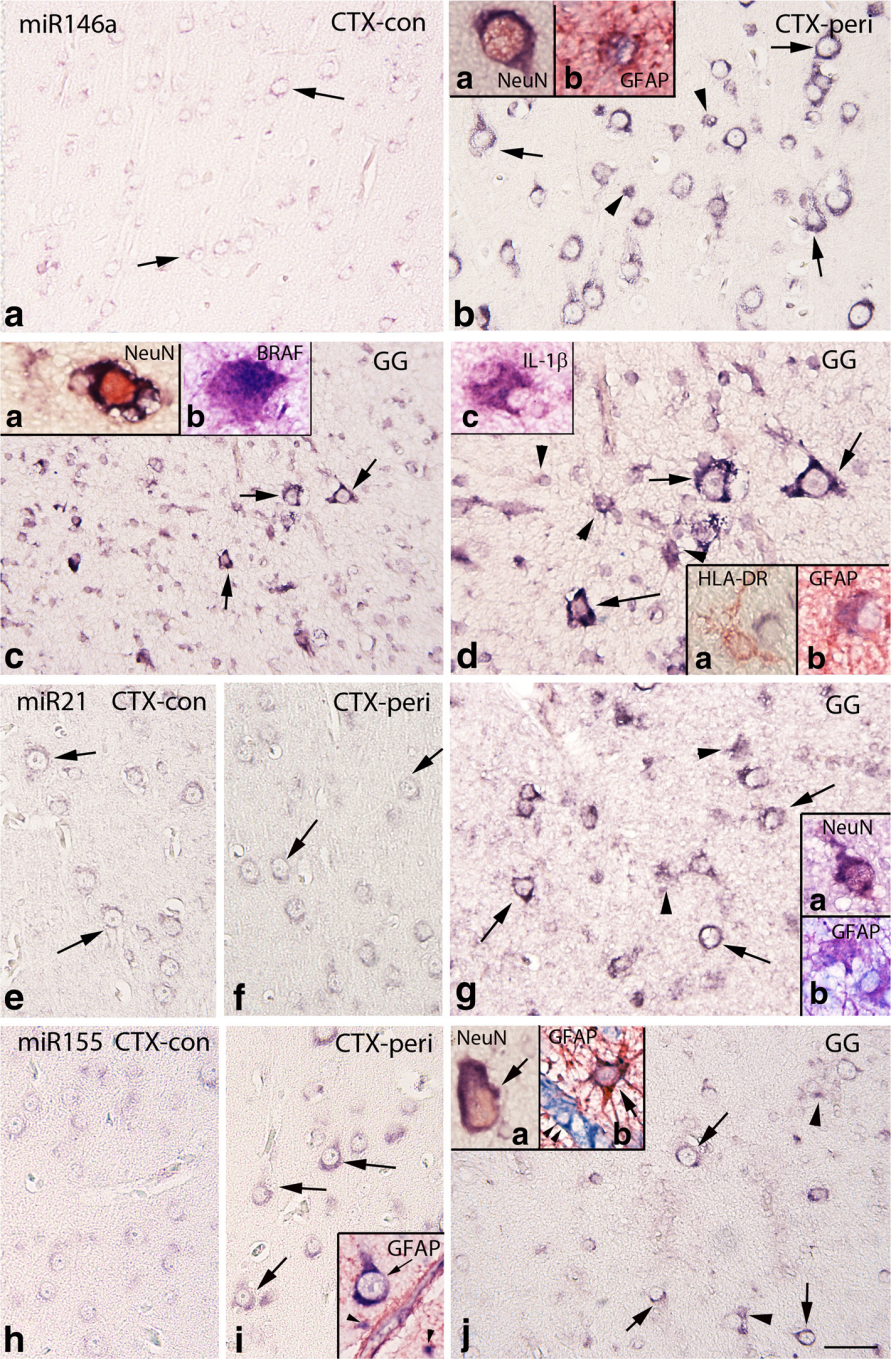
In situ hybridization of miR146a, miR21, and miR155 expression in control, peritumoral cortex, and GG. **a**–**d** miR146a. **a** Control cortex (−con); miR146a was expressed at low levels in neurons (*arrows*) and was undetectable in glial cells. **b** Peritumoral cortex (CTX-peri), showing miR146a expression in neurons (*arrows*) and glial cells (*arrowheads*). **c**–**d** GG; miR146a was expressed in both the neuronal (*arrows*) and the glial (*arrowheads* in **d**) tumor components. Inserts (*a*) in (**b)** and (**c)** show expression of miR146a in a neuron (NeuN positive, *red*); inserts (*b*) in (**b)** and in (**d)** show colocalization (*purple*) of miR146a with GFAP (*red*). Insert (*b*) in **c** shows colocalization (*purple*) of miR146a with BRAF (*red*). Insert (*a*) in (**d)** shows absence of colocalization with HLA-DR (microglia, *red*). Insert (*c*) in (**d)** shows colocalization (*purple*) of miR146a with IL-1β (*red*). **e**–**g** miR21. In both control (**e**) and peritumoral cortex (**f**), miR21 was expressed at low levels in neurons (*arrows*) and was undetectable in glial cells. **g** GG, showing miR21 expression in neurons (*arrows*) and glial cells (*arrowheads*). Insert (*a*) in (**g)** shows expression of miR21 in a neuron (NeuN positive, *red*); insert (*b*) shows expression of miR21 in astrocytes (GFAP positive, *red*). **h**–**j** miR155. **h** Control cortex, showing low expression of miR155. In peritumoral cortex (**i**), moderate expression was observed in neurons (*arrows*). **j** GG, showing miR155 expression in neurons (*arrows*) and glial cells (*arrowheads*). Insert in **i** shows expression of miR155 in a neuron (*arrow*) and astrocytes (GFAP positive, *red*; *arrowheads*) around a positive blood vessel. Insert (*a*) in **j** shows expression of miR155 in a neuron (NeuN positive, *red*); insert (*b*) shows expression of miR155 in an astrocyte (GFAP positive, *red*; *arrow*) around a positive blood vessel (*arrowheads*); scale bar in (**j)**: (**a**, **c)** 150 μm; (**b**, **e**–**j)** 80 μm; (**d)** 40 μm

**Fig. 4:**
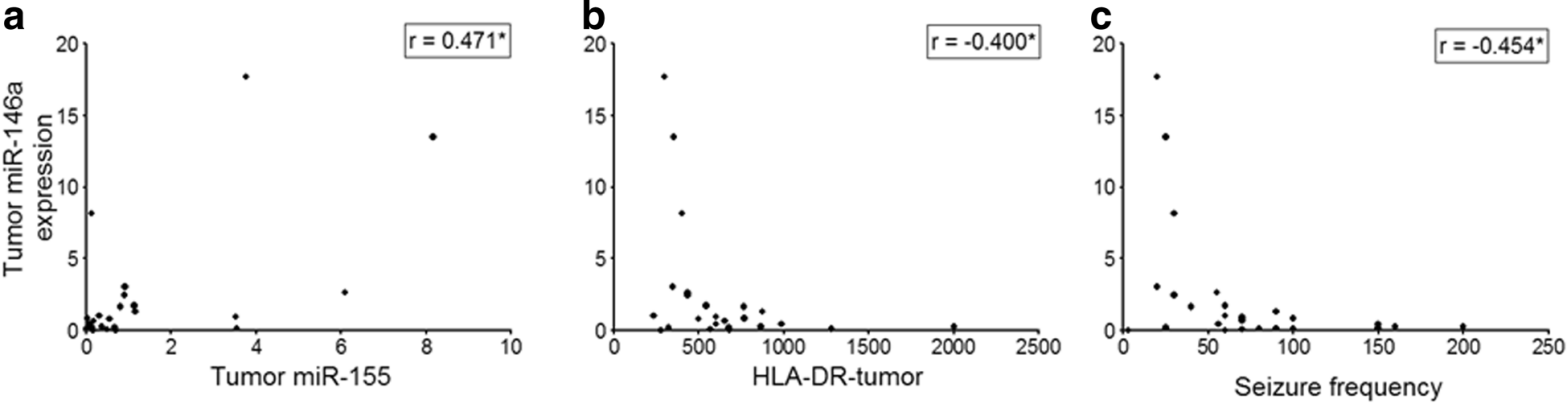
miR146a expression in GG: correlation with miR155, number of HLA-DR-positive cells and seizure frequency. Scatter plots showing the significant correlation between tumor miR146a expression and (**a)** tumor miR155 expression; (**b)** number of HLA-DR-positive cells within the tumor; (**c)** pre-operative seizure frequency; *r* = Spearman’s rank correlation coefficient. *p < 0.05; **p < 0.01

The cellular distribution of miR21, miR146a, and miR155 in GG and peritumoral cortex was investigated using in situ hybridization. In control cortex, miR146a was expressed at low levels in neuronal cells but was undetectable in glial cells (Fig. 3a). miR146a expression was increased in both tumor and peritumoral cortex. We detected miR146a expression in cells with typical astroglia morphology and in dysplastic neurons (Fig. 3b–d). Double labeling confirmed miR146a expression in NeuN- and GFAP-positive cells, whereas no detectable expression was observed in HLA-DR-positive cells of the microglial/ macrophage lineage (Fig. 3b–d); miR146a was also detected in BRAF V600E and IL-1β-positive cells (Fig. 3d). miR146a was positively associated with miR155 expression within the tumor (*r* = 0.471, *p* = 0.018; Fig. 4a). The tumor miR146a expression was negatively correlated with the density of activated microglial cells (*r* = −0.400, *p* = 0.043; Fig. 4b) and CD34 expression (*r* = −0.541, *p* = 0.037; not endothelial cells). A positive correlation was observed between miR146a expression in the peritumoral cortex and GFAP IRS (*r* = 0.695, *p* = 0.004).

In both control and peritumoral cortex, miR21 was mainly detected in neuronal cells (Fig. 3e, f). In GG, the expression of miR21 was increased and it was detected in both neuronal and glial cells, as confirmed by double-labeling experiments (Fig. 3g). A positive correlation was observed between miR21 expression in GG and GFAP IRS (*r* = 0.396, *p* = 0.045). Expression of miR155 in control cortex was mainly observed in neuronal cells (Fig. 3h). In peritumoral cortex and within the tumor, miR155 was expressed in both neurons and glial cells (NeuN- and GFAP-positive cells; Fig. 3i, j); we also observed expression of miR155 in blood vessels (peritumoral cortex and tumor; Fig. 3i, j).

### Regulation of miR21, miR146a, and miR155 expression by IL-1β in cell culture

**Fig. 5:**
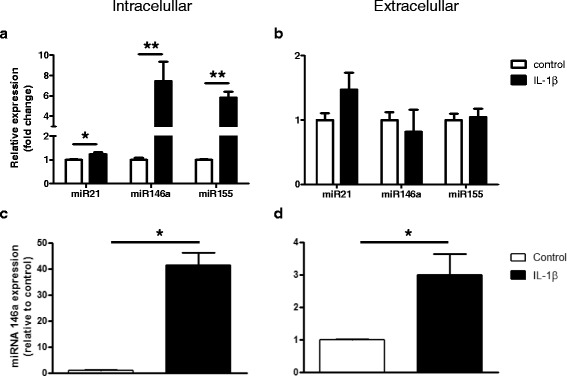
miRNAs expression levels after exposure to IL-1β in culture. Quantitative real-time PCR. Cellular (**a**) and extracellular (**b**) levels of miR21, miR146a, and miR155 in U373 glioblastoma cells 24 h after exposure to IL-1β (10 ng/ml). Cellular (**c**) and extracellular (**d**) levels of miR146a in human fetal astrocytes in culture 24 h after exposure to IL-1β (10 ng/ml). Data are expressed relative to the levels observed in unstimulated cells and are mean ± SEM from two cultures (*p < 0.05 compared to control). miRNAs expression was normalized to that of the U6B small nuclear RNA gene (Rnu6B) or to that of miR23a for the intra- and extracellular fractions, respectively

In the present study, we used both U373 glioblastoma cells and fetal astrocytes in culture to examine the effect of IL-1β on miR21, miR146a, and miR155 expression. qPCR analysis demonstrated that exposure to IL-1β increased miR21, miR146a, and miR155 expression in U373 cells (Fig. 5a). Also, a tendency towards increased extracellular miR21 levels was observed (Fig. 5b). In fetal astrocytes, we observed a prominent upregulation of both intracellular and extracellular miR146a (Fig. 5c, d).

### miR21, miR146a, and miR155 expression and clinical features in GG

We found no statistically significant association between miR21, miR146a, and miR155 expression and clinical features, such as gender, age at surgery, location of the tumor, or duration of epilepsy. However, a negative correlation was observed between tumor and peritumoral miR146a expression and the pre-operative seizure frequency (GG: *r* = −0.454, *p* = 0.02; Fig. 4c; peritumoral cortex: *r* = −0.538, *p* = 0.039).

### miRNA target expression in GG

We evaluated the expression and cellular distribution of PDCD4 (target of miR21), TRAF6 and IRAK1 (target of miR146a), and SHIP1 (target of miR155) in GG by immunocytochemistry in the large cohort of samples (FFPE, *n* = 26). Both PDCD4 and TRAF6 were expressed in the astroglial component of the tumor (Additional file 2: Figure S2A, B), and a negative correlation was observed between the PDCD4 IRS and the miR21 (*r* = −0.817, *p* = <0.001), as well as between the TRAF6 IRS and the miR146a (*r* = −0.498, *p* = < 0.05). Expression of TRAF6 was positively associated with the pre-operative seizure frequency (*r* = 0.651, *p* = < 0.01), the density of activated microglial cells (*r* = 0.522, *p* = < 0.01), and the IL-1β IRS (*r* = 0.515, *p* = < 0.01). SHIP1 and IRAK1 were expressed in both neuronal and glial cells (Additional file 2: Figure S2C, D). There was a negative correlation between miR155 and SHIP1 protein expression in glial cells in both tumor (*r* = −0.439, *p* = <0.05) and in peritumoral cortex (*r* = −0.699, *p* = <0.05). IRAK1 expression was negatively correlated with miR146a (*r* = −0.560, *p* = <0.05). No significant correlations were observed between miR155 and neuronal SHIP1, as well as between miR146a and neuronal IRAK1.

Western blot analysis performed in a small cohort of GG (*n* = 8) showed an increased expression of TRAF6 compared to controls and variable expression of IRAK1 and IRAK2 (Additional file 3: Figure S3); only a tendency towards a negative correlation of miR146a with IRAK2 protein expression was observed (*r* = −0.587, *p* = 0.07).

Evaluation of mRNA expression levels of downstream targets of miR21 (PTEN, PDCD4, and MEF2C), miR146a (IRAK1, IRAK2 and TRAF6, ERBB4, NOTCH1, and NUMB), and miR155 (SHIP1) showed significant down-regulation of PTEN and NUMB mRNA expression in GG compared to controls (data not shown; *p* < 0.001). We also detected a negative correlation between PTEN mRNA and the miR21 (*r* = −0.517, *p* = <0.04) and a tendency towards a negative correlation between NUMB mRNA and miR146a (*r* = −0.497, *p* = 0.0501).

### Microglial activation, IL-1β, and clinical features in GG

The density of activated microglial cells (HLA-DR-positive cells) within the tumor was positively correlated with the pre-operative seizure frequency (0.834, *p* < 0.001), the duration of epilepsy (0.631, *p* = 0.001), and the age at surgery (0.521, *p* = 0.006); positive correlation was also observed with the IL-1β IRS in both tumor and peritumoral cortex (0.838, *p* < 0.001; 0.535, *p* = 0.04); Additional file 4: Figure S1A–C.

Expression of IL-1β in both tumor and peritumoral cortex was positively associated with the pre-operative seizure frequency (GG 0.855, *p* < 0.001; peritumoral ctx 0.579, *p* = 0.02) and the duration of epilepsy (GG 0.441, *p* = 0.02; peritumoral ctx 0.669, *p* = 0.006); Additional file 4: Figure S1D–F.

## Discussion

GGs represent a major cause of medically intractable epilepsy in young patients; however, the cellular mechanisms underlying the intrinsic and high epileptogenicity of these lesions remain to be fully investigated [1, 2].

Over the past decade, an increasing number of experimental studies in animal models, as well as clinical and neuropathological observations, obtained in human brain specimens from various drug-resistant types of epilepsy (including GGs), demonstrated the relevance of inflammation in the pathophysiology of epilepsy (for reviews see [11, 14, 45–47]). In particular, the IL-1R/TLR proinflammatory pathway has been shown to critically contribute to seizure development, and targeting proinflammatory pathways has been suggested as strategy to obtain disease-modifying effects (i.e., decreased frequency and severity of chronic seizure and reduced comorbidity; [46, 48, 49]). Induction of both innate and adaptive immune system with prominent activation of proinflammatory proictogenic pathways (such as of the IL-1R/TLR) has also been observed in GGs ([7–9, 15, 50] present study). In this study, we provide additional evidence of the activation of the Toll-like and interleukin-1 receptor (TIRs) signaling, showing increased expression of downstream signaling molecules (IRAK1 and TRAF6) of the TIR pathway.

Interestingly, the density of activated microglial cells has been previously shown to be positively correlated with the frequency of seizures prior to surgical resection and the duration of epilepsy [15]. This observation has been confirmed in the present cohort, showing a positive correlation between the IL-1β expression and pre-operative seizure frequency, duration of epilepsy, as well as TRAF6 expression. A positive correlation was also observed between TRAF6 and the pre-operative seizure frequency, as well as the density of activated microglial cells.

Since several inflammatory pathways have been shown to be activated in concert in GGs [8], it is important to identify and evaluate upstream master regulators (such as miRNAs) of key proinflammatory/proictogenic pathways. In the present study, we investigated the expression and cellular distribution of miR21, miR146, and miR155, three miRNAs involved in the regulation of IL-1R/TLR proinflammatory/proictogenic pathway in a large cohort of GGs. Our findings provide evidence of a differential regulation of these three miRNAs both within the tumor and in the peritumoral region, supporting their potential active role in the clinical behavior of the tumor. The cell-specific distribution of these miRNAs in relation with the epileptogenicity of the tumor is discussed below.

### miR21 expression and distribution in GG and peritumoral cortex

Expression of miR21 has been observed in both tumor and peritumoral cortex with a tendency towards increased expression in the tumor compared to both control and peritumoral cortex. Recent studies indicate expression of this miRNA in both neuronal and glial cells [26, 51–53]. However, data about miR21 functions and cellular distribution in human brain are still limited. We observed neuronal expression in both control and peritumoral cortex, whereas within the tumor, miR21 expression was detected in both dysplastic neurons and tumor astrocytes.

miR21 has recently emerged as one of the important dysregulated miRNAs in many pathological conditions, including cardiovascular disease, brain injury, ischemia, and in a variety of human neoplastic disorders, representing the most commonly and strongly upregulated miRNA in high-grade gliomas [54, 55]. Among the various targets of miR21 are tumor suppressors such as phosphatase and tensin homolog (PTEN), programmed cell death 4 (PDCD4), components of the p53 pathway, or transforming growth factor-β (TGF-β) signaling, resulting in increase of cell proliferation, survival and migration/invasion in tumors [54–56]. In our retrospective study, we could not determine whether the expression levels of this miRNA within the tumor would influence local recurrence on malignant transformation and evaluation of its role in the regulation of astrocytes proliferation and differentiation is still in progress. However, we observed a negative correlation between the expression of tumor PTEN mRNA, as well as of PDCD4 protein in tumor astrocytes and the level of tumor miR21, supporting the link between miR21 and these targets. The positive correlation observed between miR21 and the expression of tumor GFAP supports the role of astrocytes as targets of regulation and as a source of miR21.

Recently, miR21 has been shown to be upregulated in animal models of temporal lobe epilepsy (TLE) after induction of status epilepticus (SE) [26, 57]. In particular, we have shown a significant increase of this miRNA in three different hippocampal regions during the latent period preceding the onset of seizure [26] and associated with increased levels of proinflammatory cytokines, such as IL-1β [58]. miR21 can be induced by NF-κB and has been suggested to act as negative-feedback regulator of Toll-like receptor signaling via targeting of the proinflammatory tumor suppressor *PDCD4* ([59]; for review see [31, 32, 55, 60]). However, in our study, only modest increase was observed in glioma cells in response to IL-1β and we did not find significant association between miR21 expression and clinical features, such as pre-operative seizure frequency or duration of epilepsy.

In addition, miR21 (as well as miR155) has been recently shown to be upregulated in focal cortical dysplasia (FCD) tissue samples [61] and in cortical tubers of patients with tuberous sclerosis complex (TSC; van Scheppingen et al., unpublished observations), suggesting an epigenetic regulation of the mammalian target of rapamycin (mTOR) signaling pathway, which is also activated in GGs [10]. Thus, a potential role for this miRNA in neuronal dysfunction and mTOR activation can be considered and deserves further investigation.

### miR146a expression and distribution in GG and peritumoral cortex

We confirmed, in a large tumor cohort, the previously observed increased expression of miR146a in GG [25]. In situ hybridization showed increased expression of this miRNA in tumor astrocytes and colocalization with IL-1β was observed. Accordingly, IL-1β represents a major inducer of this miRNA in both human astrocytes and glioma cells ([25]; present data). Similar to miR21, miR146a has also been shown to increase in reactive astrocytes at 1 week after SE, which corresponds to the time of maximal astroglial activation [26, 62] and upregulation in astrocytes has also been reported in human TLE [62, 63]. Recent studies support the function of this miRNA as a key negative-feedback regulator of the astrocyte-mediated inflammatory response (for reviews see [31, 32]). In particular, miR146a overexpression has been shown to reduce major inflammatory molecules induced by IL-1β [25]. Accordingly, we observed a negative correlation between the level of tumor miR146a and the expression of TRAF6, a downstream signaling molecule of the TIR signaling pathway that has previously been shown to be a miR146 target. Interestingly, the expression of TRAF6 was positively associated with the pre-operative seizure frequency.

In our cohort, the tumor miR146a expression was negatively correlated with the density of activated microglial cells. Moreover, we also observed a negative correlation between tumor miR146a expression and the pre-operative seizure frequency. Interestingly, alterations of miR146a brain level have recently shown to modify acute seizures in mice and to affect brain inflammation triggered by activation of the proictogenic IL-1R/TLR signaling pathway ([64]; unpublished observation), supporting the potential for targeting this miRNA as strategy for modulating inflammatory pathways and seizure activity.

Secreted miRNAs may represent a new form of intercellular communication, acting as signaling molecules [65–67] and have been also shown to play a key role in the biology of the tumor microenvironment [68]. In the present study, we provide evidence of release of miR146a upon stimulation with IL-1β in fetal human astrocytes in culture.

Surprisingly, miR146a is also expressed within the dysplastic and immature neuronal cell components of GG, and we observed a negative correlation between the levels of this miRNA and the expression of the precursor cell marker, CD34 [69]. Interestingly, miR146a has been recently shown to modulate neural stem cell proliferation and differentiation, and induction of this miRNA in gliomas has been suggested to represent a negative-feedback mechanism to restrict tumor growth [70]. Such inhibitory function of miR146a on gliomas has been attributed to the down-regulation of the key neural stem cell factor NOTCH1 [70]. However, another key target of mir146a is represented by NUMB [71, 72] that has been shown to regulate cell fate and growth, acting as tumor suppressor [73–75]. Interestingly, we observed a down-regulation of NUMB in GG and a tendency towards a negative correlation with miR146a. Thus, further study is required to determine whether the expression levels of this miRNA would differentially regulate proliferation and/or differentiation of GG glial and neuronal components. Moreover, since a mutation of the BRAF oncogene is frequently observed in GGs [39, 50, 76, 77], its relationship with the expression of miR146a represents another aspect that deserves attention. Accordingly, a recent study shows that miR146a, upregulated by oncogenic BRAF, promotes the initiation and progression of melanoma cells through down-regulation of NUMB [78]. In our study, we observed a colocalization with the BRAF V600E-mutated protein in dysplastic neurons.

Increased expression of miR146a has been also observed in the peritumoral cortex with expression in reactive astrocytes, and a positive correlation was observed between miR146a levels and the expression of GFAP. Both clinical and experimental studies support the epileptogenicity of the peritumoral zone, which may contribute to the generation and propagation of seizure activity [79–81]. The negative correlation observed between peritumoral miR146a expression and the pre-operative seizure frequency support the potential contribution of this miRNA to the peritumoral epileptogenic network.

### miR155 expression and distribution in GG and peritumoral cortex

miR155 has been extensively studied as a cancer-associated miRNA and has been shown to be over-expressed in various types of cancers (for review see [30, 56]). In particular, it has been recently shown to promote glioma cell proliferation via the regulation of MXI1 [82] and GABA receptors [83]. In our study, miR155 has been observed in both tumor and peritumoral cortex, with increased expression in peritumoral tissue compared to the tumor. In both tumor and peritumoral cortex, we observed astroglial expression of miR155. Accordingly, this miRNA has been shown to be expressed in human astrocytes and upregulated in response to cytokines and TLR ligands [84]. Increased expression of this miRNA has been also reported in multiple sclerosis lesions [85]. In our study, we showed induction of miR155 in response to IL-1β in glioma cells. Although miR155 has been shown to exert both positive and negative effects on the NF-kB signaling proteins ([86]; reviewed in [30, 31]), in vitro studies support the positive role in the induction of proinflammatory genes in human astrocytes [84]. Thus, the expression of this miRNA observed in the peritumoral area may counteract the anti-inflammatory function of miR146. miR21 has been shown to play an important role in the regulation of miR155 expression by promoting the expression of IL-10, a key negative regulator of miR155 expression [30, 31]. Interestingly, low levels of expression were detected for miR21 in the peritumoral cortex.

In both tumor and peritumoral cortex, expression of miR155 has been also detected in endothelial cells. Accordingly, miR155 has been shown to regulate endothelial cell functions and angiogenesis and negatively affect blood–brain barrier function during neuroinflammation [87–90].

Recently, miR155 has been shown be upregulated in animal models of TLE [91, 92], as well as in FCD tissue samples [61] and in TSC (van Scheppingen et al., unpublished observations). In the present study, we did not find a significant association between miR155 expression and clinical features, such as pre-operative seizure frequency or duration of epilepsy.

An issue to be considered in the interpretation of these data is represented by the possible influence of anti-epileptic drugs, such as valproate and phenobarbital, on miRNAs expression [93, 94].

In conclusion, this study provides information on the cellular distribution and expression of three inflammation-related microRNAs in GGs. Our results indicate a differential regulation of these miRNA involving also the epileptogenic peritumoral region. In particular, the negative correlation observed between miR146a expression, the density of activated microglial cells, and the pre-operative seizure frequency support the potential role of miR146a in regulating the clinical behavior of these epileptogenic lesions, pointing to this upstream regulator of proinflammatory/proictogenic pathways as attractive target for further preclinical studies in drug-resistant epilepsies.

## Additional files

Additional file 1: Table S1. Real-time quantitative PCR analysis (qPCR; microRNA targets).

Additional file 2: Figure S2. PDCD4, TRAF6, SHIP1, and IRAK1 expression in GG. Representative photomicrographs of PDCD4 (A), TRAF6 (B), SHIP1, (C) and IRAK1 (D) IR in GG showing expression in tumor astrocytes (arrows); arrowhead in C shows neuronal expression of SHIP1 (C); insert in D: IRAK1 positive neuron (D). Sections were counterstained with hematoxylin. A–F: scale bar: 40 μm.

Additional file 3: Figure S3. TRAF6, IRAK1, and IRAK2 protein expression. Western blot analysis. A–C: representative immunoblot of total homogenates from GG densitometric analysis: values (optical density units, O.D.) are mean ± SEM (*n* = 8), relative to the optical density of β-actin; *p < 0.05, compared to controls (*n* = 6). (PPTX 72 kb)

Additional file 4: Figure S1. HLA-DR-positive cells and IL-1β expression in GG: correlation with clinical variables. A–C: scatter plots showing the significant correlation between HLA-DR-positive cells and (A) tumor IL-1β immunoreactivity score (IRS; insert in A shows IL-1β IR within the tumor); (B) pre-operative seizure frequency and (C) duration of epilepsy. D–F: Scatter plots showing the significant correlation between tumor and (D) peritumoral IL-1β IRS (insert in D shows IL-positive astrocytes within the peritumoral cortex); (E) pre-operative seizure frequency and (F) duration of epilepsy; *r* = Spearman’s rank correlation coefficient, *p < 0.05, **p < 0.01.
