# Supplementary material for: Differential expression and clinical significance of three inflammation-related microRNAs in gangliogliomas
Source: J Neuroinflammation. 2015 May 20;12:97. doi: 10.1186/s12974-015-0315-7 (PMC4446114; doi:10.1186/s12974-015-0315-7)
Supplement: Supplementary file 1 — Real-time quantitative PCR analysis (qPCR; microRNA targets). [file 12974_2015_315_MOESM1_ESM.doc]

**Real- time Quantitative PCR Analysis (qPCR; microRNA targets)**

Five nmol oligo dT primers were annealed to 5 µg total RNA in a total volume of 25 µl, by incubation at 72 °C for 10 min, and cooled to 4°C. Reverse transcription was performed by the addition of 25 µl RT-mix, containing: First Strand Buffer (Invitrogen-Life Technologies), 2 mM dNTPs (Pharmacia, Germany), 30 U RNAse inhibitor (Roche Applied Science, Indianapolis, IN, USA) and 400 U M-MLV reverse transcriptase (Invitrogen - Life Technologies, The Netherlands). The total reaction mix (50 µl) was incubated at 37 °C for 60 min, heated to 95 °C for 10 min and stored at -20°C until use. PCR primers (Eurogentec, Belgium) were designed using the Universal Probe Library of Roche ([https://www.roche-applied-science.com](https://www.roche-applied-science.com/)) on the basis of the reported mRNA sequences (Supplementary Table 1). For each PCR, a mastermix was prepared on ice, containing per sample: 1 µl cDNA, 2.5 µl of SensiFAST SYBR no-ROX mix (Bioline Reagents Ltd, London, UK), 0.4 µM of both reverse and forward primers. The final volume was adjusted to 5 µl with PCR grade water. The LightCycler® 480 Real-Time PCR System (Roche-applied-science) was used with a 384-multiwell plate format. The cycling conditions were carried out as follows: initial denaturation at 95°C for 2 min, followed by 45-50 cycles of denaturation at 95°C for 5 s, annealing at 65°C for 10 s and extension at 72°C for 15 s. The fluorescent product was measured by a single acquisition mode at 72°C after each cycle. For distinguishing specific from non-specific products and primer dimers, a melting curve was obtained after amplification by holding the temperature at 65°C for 1 min followed by a gradual increase in temperature to 95°C at a rate of 2.5°C/s, with the signal acquisition mode set to continuous. Quantification of data was performed using the computer program LinRegPCR in which linear regression on the Log(fluorescence) per cycle number data is applied to determine the amplification efficiency per sample The starting concentration of each specific product was divided by the starting concentration of reference gene (EF1a) and this ratio was compared between patient/control groups.

Western blot analysis

The frozen specimens were homogenized in lysis buffer containing 10 mM Tris (pH 8.0), 150 mM NaCl, 10% glycerol, 1% NP-40, 0.4 mg/ml Na-orthovanadate, 5 mM EDTA (pH 8.0), 5 mM NaF and protease inhibitors (cocktail tablets, Roche Diagnostics, Mannheim, Germany). Protein content was determined using the bicinchoninic acid method. For electrophoresis, equal amount of proteins (50 μg/lane) were separated by sodium dodecylsulfate-polyacrylamide gel electrophoresis (SDS-PAGE, 10% acrylamide). Separated proteins were transferred to nitrocellulose paper by electroblotting for 1 h and 30 min (BioRad, Transblot SD, Hercules, CA). After blocking for 1 h in TBST (20 mM Tris, 150 mM NaCl, 1 % Tween, pH 7.5)/5% non-fat dry milk, blots were incubated overnight at 4 °C with rabbit anti-IRAK1 (1:1000, SC-7833, Santa Cruz Biotechnology, Heidelberg, Germany), rabbit anti-IRAK2 (1:500, B22, Santa Cruz Biotechnology, Heidelberg, Germany), rabbit anti-TRAF6 (1:1000, EP591Y, Abcam, Cambridge, UK) or mouse anti-β-Tubulin (1:1000, clone D66, Sigma, St. Louis, MO, USA). After several washes in TBST, the membranes were incubated in TBST / 5% non-fat dry milk, containing the goat anti-rabbit or rabbit anti-mouse antibodies coupled to horse radish peroxidase (1:2500; Dako, Denmark) for 1h. After washes in TBST, immunoreactivity was visualized using ECL PLUS western blotting detection reagent (GE Healthcare Europe, Diegen, Belgium). For the quantification of the blots, the band intensities were measured densitometrically by using the Scion Image for Windows (beta 4.02) image-analysis software. A ratio of the integrated band density (IntDen) of the protein of interest to the IntDen of the reference protein was used to normalize band intensities.

| **Gene** | **Forward primer** | **Reverse primer** | **Amplicon size**  **(nt)** |
| --- | --- | --- | --- |
| IRAK1 | gcccgaggagtacatcaaga | ctctgaccagccaaggtctc | 93 |
| IRAK2 | cctcctctgaggcctgtgt | tgatctcaatttgccacgaa | 72 |
| TRAF6 | tggcattacgagaagcagtg | tggacatttgtgacctgcat | 95 |
| PDCD4 | tggaaagcgtaaagatagtgtgtg | ttctttcagcagcatatcaatctc | 91 |
| SHIP1 | gtccaaggatggttctgagg | gtgacttaatgagctgcaggatt | 65 |
| ERBB4 | aggagtgaaattggacacagc | tctcggtatacaaactggtttcc | 71 |
| MEF2C | tgatcagcaggcaaagattg | tggacactgggatggagact | 72 |
| NOTCH1 | ctgcctgtctgaggtcaatg | tcacagtcgcacttgtaccc | 90 |
| PTEN | gcacaagaggccctagatttc | cgcctctgactgggaatagt | 77 |
| NUMB | gttgtcatgggggaggtg | ttgcttaagcctcaaatctgc | 105 |
| EF1A | atccacctttgggtcgcttt | ccgcaactgtctgtctcatatcac | 51 |
