# Supplementary figures and images for: Differential expression and clinical significance of three inflammation-related microRNAs in gangliogliomas
Source: J Neuroinflammation. 2015 May 20;12:97. doi: 10.1186/s12974-015-0315-7 (PMC4446114; doi:10.1186/s12974-015-0315-7)

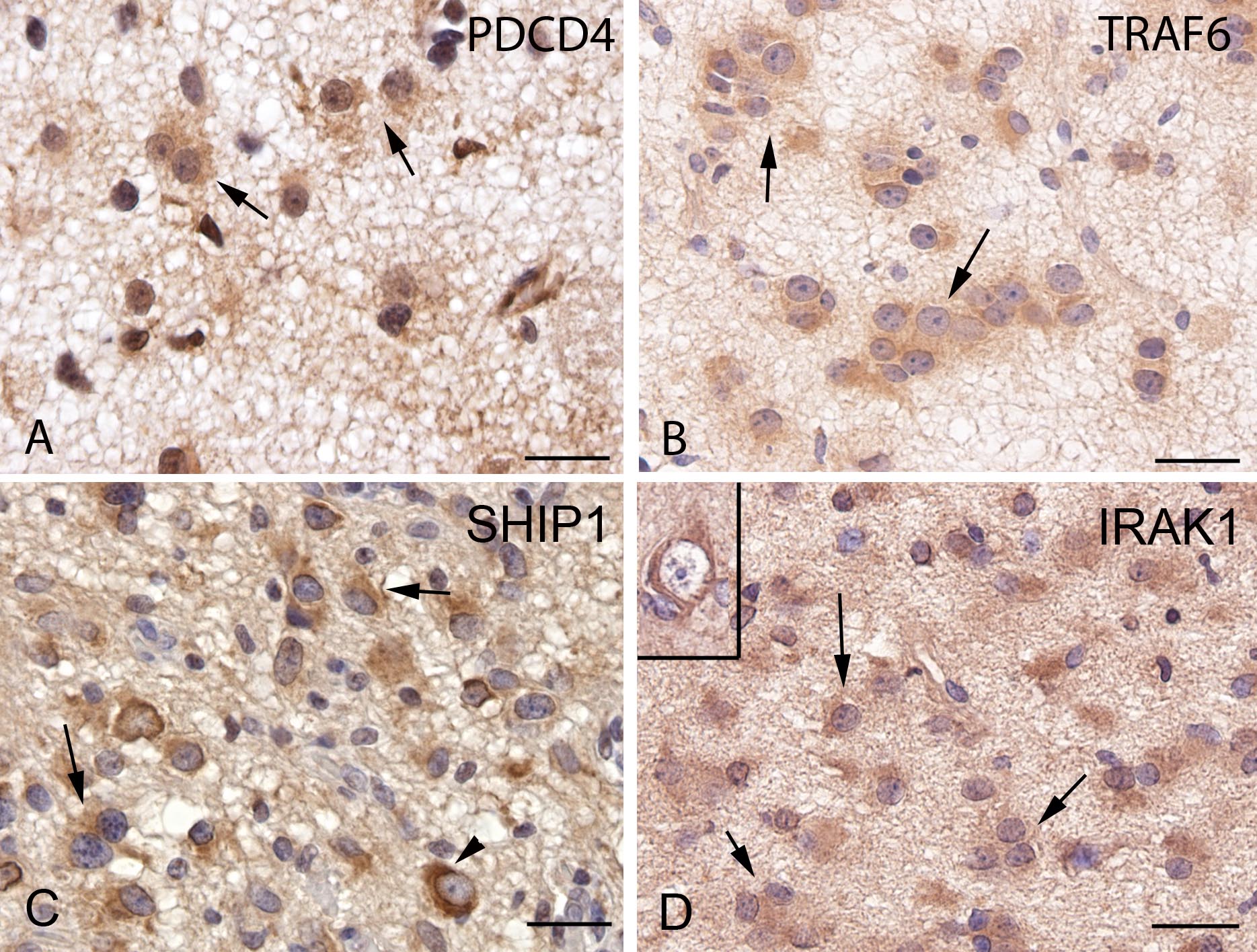

Supplement: Supplementary file 2 — PDCD4, TRAF6, SHIP1, and IRAK1 expression in GG. Representative photomicrographs of PDCD4 (A), TRAF6 (B), SHIP1, (C) and IRAK1 (D) IR in GG showing expression in tumor astrocytes (arrows); arrowhead in C shows neuronal expression of SHIP1 (C); insert in D: IRAK1 positive neuron (D). Sections were counterstained with hematoxylin. A–F: scale bar: 40 μm. [file 12974_2015_315_MOESM2_ESM.jpg]

## Slide 1
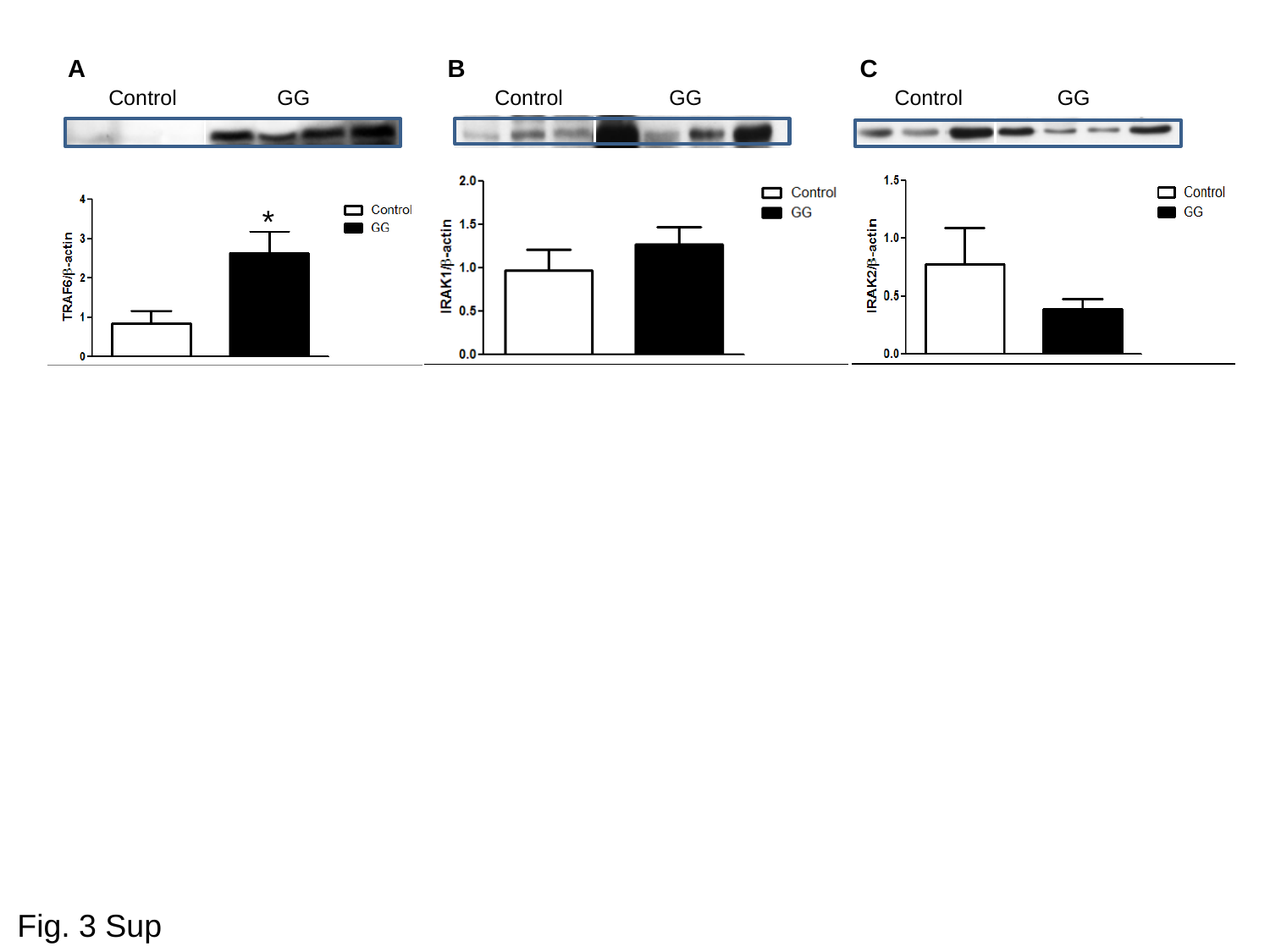

A
B
C
Control GG
 Control GG
 Control GG
*
Fig. 3 Sup

Supplement: Supplementary file 3 — TRAF6, IRAK1, and IRAK2 protein expression. Western blot analysis. A–C: representative immunoblot of total homogenates from GG densitometric analysis: values (optical density units, O.D.) are mean ± SEM (n = 8), relative to the optical density of β-actin; *p < 0.05, compared to controls (n = 6). (PPTX 72 kb) [file 12974_2015_315_MOESM3_ESM.pptx]

## Slide 1
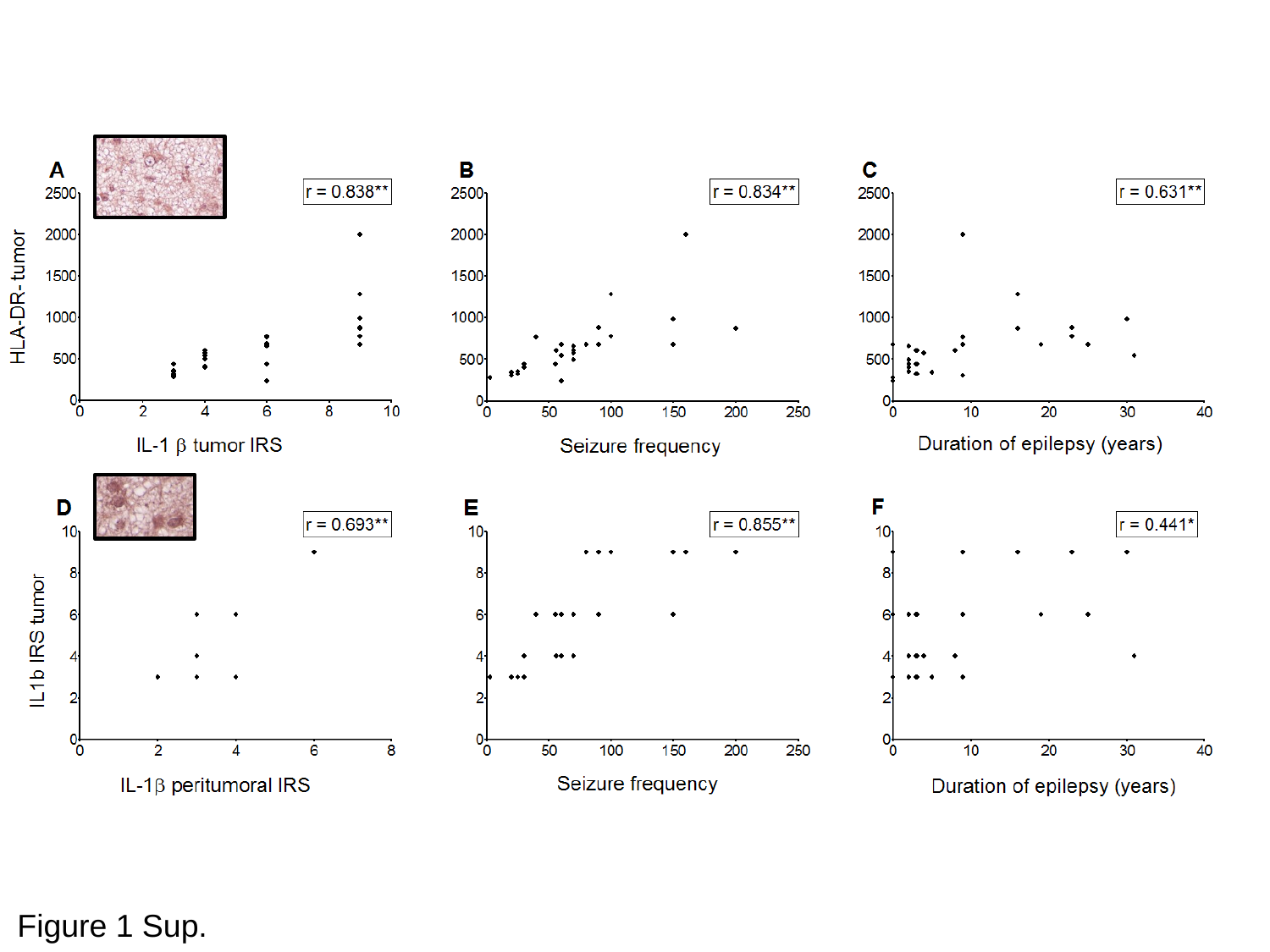

Figure 1 Sup.

Supplement: Supplementary file 4 — HLA-DR-positive cells and IL-1β expression in GG: correlation with clinical variables. A–C: scatter plots showing the significant correlation between HLA-DR-positive cells and (A) tumor IL-1β immunoreactivity score (IRS; insert in A shows IL-1β IR within the tumor); (B) pre-operative seizure frequency and (C) duration of epilepsy. D–F: Scatter plots showing the significant correlation between tumor and (D) peritumoral IL-1β IRS (insert in D shows IL-positive astrocytes within the peritumoral cortex); (E) pre-operative seizure frequency and (F) duration of epilepsy; r = Spearman’s rank correlation coefficient, *p < 0.05, **p < 0.01. [file 12974_2015_315_MOESM4_ESM.pptx]
